# A new primer construction technique that effectively increases amplification of rare mutant templates in samples

**DOI:** 10.1186/s12896-019-0555-1

**Published:** 2019-08-23

**Authors:** Jr-Kai Huang, Ling Fan, Tao-Yeuan Wang, Pao-Shu Wu

**Affiliations:** 1 Department of Pathology, Mackay Memorial Hospital, Taipei, Taiwan; 2 Department of Nuclear Medicine, Chang Gung Memorial Hospital, Taoyuan, Taiwan; 3 Mackay Junior College of Medicine, Nursing, and Management, Taipei, Taiwan

**Keywords:** Cancer, Mutation, cfDNA

## Abstract

**Background:**

In personalized medicine, companion diagnostic tests provide additional information to help select a treatment option likely to be optimal for a patient. Although such tests include several techniques for detecting low levels of mutant genes in wild-type backgrounds with fairly high sensitivity, most tests are not specific, and may exhibit high false positive rates. In this study, we describe a new primer structure, named ‘stuntmer’, to selectively suppress amplification of wild-type templates, and promote amplification of mutant templates.

**Results:**

A single stuntmer for a defined region of DNA can detect several kinds of mutations, including point mutations, deletions, and insertions. Stuntmer PCRs are also highly sensitive, being able to amplify mutant sequences that may make up as little as 0.1% of the DNA sample.

**Conclusion:**

In conclusion, our technique, stuntmer PCR, can provide a simple, low-cost, highly sensitive, highly accurate, and highly specific platform for developing companion diagnostic tests.

**Electronic supplementary material:**

The online version of this article (10.1186/s12896-019-0555-1) contains supplementary material, which is available to authorized users.

## Backgroud

In personalized medicine, especially cancer therapy, companion diagnostics are tests that provide additional information to help select proper medication for each patient. To increase mutation detection sensitivity in such tests, and reduce interference from wild-type templates, several methods such as co-amplification at lower denaturation temperature (COLD) PCR [1–3], dual priming oligonucleotide (DPO)-PCR [4–6], real-time PCR [7–9], high resolution melting (HRM) analysis [10–13], next generation sequencing (NGS) [14–19], and droplet digital PCR [20–23] are used. Most of these techniques improve signal amplification and mutant sequence enrichment; however, with rising detection sensitivity, data accuracy must also be maintained, and many of these techniques fail in this regard due to the high rates of false positive or false negative results.

Direct sequencing of PCR products is highly accurate, but has low mutation detection sensitivity, being able to only detect ~ 20% of mutant alleles in a background of normal alleles [24, 25]. Although allele-specific (AS) PCR can increase detection sensitivity by using type-specific primers, false positive rates are high due to non-specific product formation [26–29]. In such cases, validation of the PCR product using sequencing is unhelpful as the primer alters the original sequences. Other signal amplification methods can detect mutations in samples with low tumor cell content, but may have high rates of false positives due to non-specific binding.

Although COLD PCR can amplify many mutations, including unknown ones, and provides higher detection sensitivity and reliable results, the technique is difficult to use when two or more genes must be detected in tandem.

The design of the tumor cell enrich methods [30–33] needs to consider the Tm value and ratio of primer and block. When the Tm value of block is too high, it is difficult to distinguish between the wild type and mutant template; If it is too low, the advantage of combining with the wild type template is lost, resulting in a decrease in sensitivity of detection.

To solve the problem of decreasing detection specificity due to increasing detection sensitivity, we present a new method, which we name ‘stuntmer PCR’. The ‘stuntmer’ is a universal primer design we have developed to detect mutations occurring within a defined region. Theoretically, several different mutations can be detected by a single stuntmer designed for a specific region. The stuntmer is so designed that when it is used as a forward primer in PCR reactions, amplification of the wild-type template is suppressed, and mutant forms of the template are selectively amplified. In stuntmer PCR, only one primer set is required to test for many mutations. Since the stuntmer sequence is the same as the reference sequence, it does not create an artificial sequence in the event of non-specific binding. Sequencing showed that mutations identified positively by stuntmer PCR were indeed correct, indicating that this PCR technique specifically enriches mutant PCR products. Furthermore, due to its adjustable detection sensitivity, stuntmer PCR is also suitable for different types of specimens, including cell-free DNA (cfDNA).

## Results

### Selective amplification of mutant templates in three different sequences

**Fig. 1:**
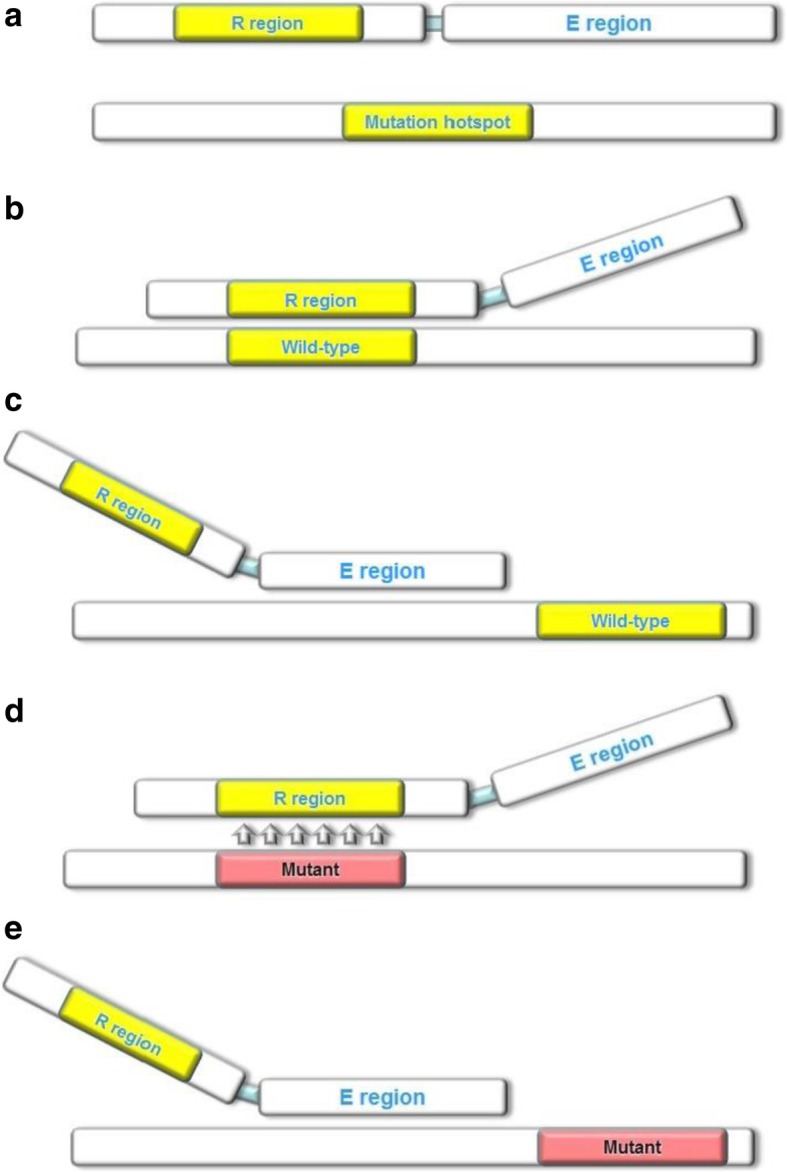
Stuntmer structure and binding conformations to wild-type and mutant templates. **a** The stuntmer primer consists of a recognition (R) region, a linker region, and an extension (E) region. The wild-type template has two sites that can bind to the stuntmer (**b**, **c**). When the R region is bound to the template, the E region remains unbound, and the wild-type template remains unamplified. The R region binding to mutant templates is unstable because of the mismatch between the two sequences. This allows the E region to bind to mutant templates (**e**), and allows amplification of mutant templates

Three stuntmers, each targeting different exons of the epidermal growth factor receptor (EGFR), namely, exon 19, exon 20 T790, and exon 21 L858/L861, were designed for this study. The sensitivities of the stuntmers were assessed using three different conditions of mutant sample prevalence: only wild-type plasmids, mixtures of wild-type:mutant plasmids in a ratio of 90:10, and mixtures of wild-type:mutant plasmids in a ratio of 99:1. The chromatograms in Fig. 1a and b show that the mutant template can be selectively amplified even when concentrations of wild-type template are ~ 100-fold higher than those of the mutant template. Stuntmer PCR does not completely inhibit wild type amplification. It can be observed from the wild-type group that even if there is no mutant type plasmid in the sample, the PCR will still perform and amplify the product. The sequencing result will also be displayed as wild (Fig. 1). From this experimental result, it can be concluded that exon 21 stuntmer’s selective amplification effect on L858R/L861Q can increase the original ratio of 1% mutation signal to 50%; the exon 20 stuntmer has a screening ability of T790 M mutation greater than 1%. Our results also demonstrate that two different mutations, L858R and L861Q, can be amplified by the same stuntmer. This clearly demonstrates that a stuntmer can inhibit wild-type template replication, thereby allowing for selective amplification of mutants in a non-sequence-specific manner.

### Comparing mutation detection sensitivities of stuntmer PCR and direct PCR methods using clinical samples

We used 1600 non-small-cell lung carcinoma samples (of which 318 were pleural effusion samples and the others were formalin-fixed, paraffin embedded (FFPE) tissue samples) to compare the mutation detection sensitivities of stuntmer PCRs and direct PCRs. After extracting DNA from the samples, we amplified the EGFR exons 19, 20, and 21, using both traditional PCR and stuntmer PCR, and sequenced the PCR products obtained. Traditional PCR was able to detect the L858R mutation in 21.88% of the samples, whereas stuntmer PCR was able to detect this mutation in 27.44% of the samples. The deletion mutation in exon 19 was detected in 20.50% of the samples via traditional PCR, whereas stuntmer PCR detected this mutation in 32.69% of the samples. The T790 M mutation was detected in 1.13% of the samples via traditional PCR, whereas stuntmer PCR detected this mutation in 3.63% of the samples. The positive predictive agreement for all three mutations was 100%, and the negative predictive agreements for L858R, the deletion in exon 19, and T790 M were 92.9, 84.7, and 97.5%, respectively (Table 1).

**Table 1:** Agreement analysis between direct PCR sequencing and stuntmer PCR

Exon 21 L858R	Traditional PCR		Total	
	Positive	Not found		
FFPE				
Stuntmer PCR				
Positive	291	56	347	
Not found	0	935	935	
Total	291	991	1282	
Pleural effusion				
Stuntmer PCR				
Positive	59	33	92	
Not found	0	226	226	
Total	59	259	318	
Total	350	1250	1600	
Positive percent agreement (95% CI)	100% (98.9, 100%)			
Negative percent agreement (95% CI)	92.9% (91.3, 94.2%)			
Overall percent agreement (95% CI)	94.4% (93.2, 95.5%)			
Exon 19 deletion	Traditional PCR			Total
	Deletion	Insertion	Not found	
FFPE				
Stuntmer PCR				
Deletion	272	0	140	412
Not found	0	0	870	870
Total	272	0	1010	1282
Pleural effusion				
Stuntmer PCR				
Deletion	56	0	55	111
Insertion	0	1	0	1
Not found	0	0	206	206
Total	56	1	261	318
Total	328	1	1271	1600
Positive percent agreement (95% CI)	100% (98.8, 100%)			
Negative percent agreement (95% CI)	84.7% (82.6, 86.5%)			
Overall percent agreement (95% CI)	87.8% (86.1, 89.3%)			
Exon 20 T790 M	Traditional PCR		Total	
	Positive	Not found		
FFPE				
Stuntmer PCR				
Positive	16	32	48	
Not found	0	1234	1234	
Total	16	1266	1282	
Pleural effusion				
Stuntmer PCR				
Positive	2	8	10	
Not found	0	308	308	
Total	2	316	318	
Total	18	1582	1600	
Positive percent agreement (95% CI)	100% (82.4, 100%)			
Negative percent agreement (95% CI)	97.5% (96.6, 98.1%)			
Overall percent agreement (95% CI)	97.5% (96.6, 98.2%)			

### Different types of mutations in the same region can be detected by the same stuntmer

**Fig. 2:**
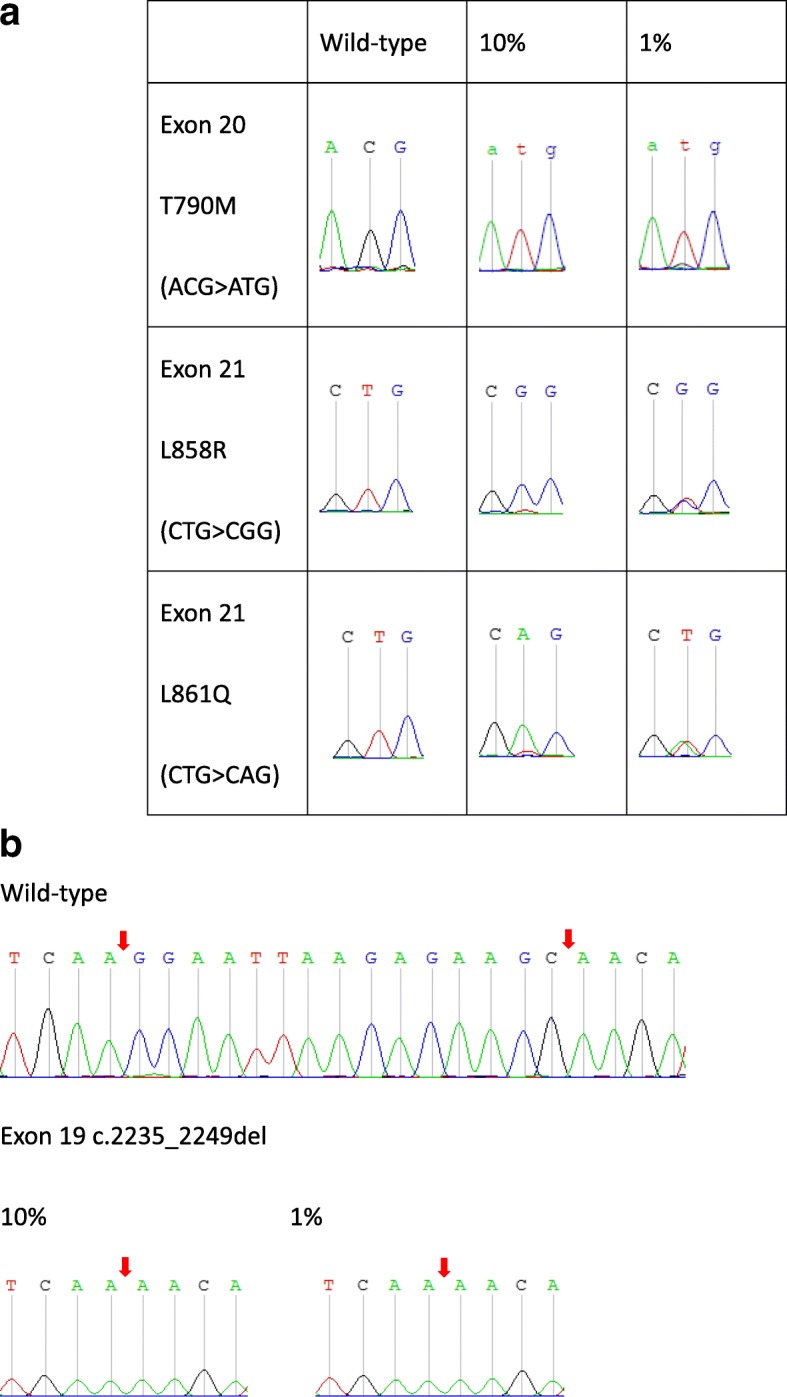
(**a**) The stuntmer primer consists of a recognition (R) region, a linker region, and an extension (E) region. The wild-type template has two sites that can bind to the stuntmer (**b**, **c**). When the R region is bound to the template, the E region remains unbound, and the wild-type template remains unamplified. The R region binding to mutant templates is unstable because of the mismatch between the two sequences (**d**). This allows the E region to bind to mutant templates (**e**), and allows amplification of mutant templates

In some cases, point mutation hotspots may overlap with insertion or deletion mutations. For example, point mutations may occur at codon 768 in exon 20 of EGFR [34] alongside several insertion mutations that may also be present between codons 761 and 775 [35]. To test if a single stuntmer can detect all these types of mutations, two mutation plasmids, c.2303G > T point mutation and c.2308_2309insCCAGCGTGG, were constructed (Fig. 2a and b). The EGFR S768 stuntmer, which was designed to detect the c.2303G > T point mutation was able to detect not only the point mutation, but also the insertion mutation even when the mutant plasmids were present at a prevalence of only 1% in the sample. The results also showed that the wild-type signal was completely suppressed in the experimental set that contained the insertion mutation. In clinical validation experiments, the stuntmer designed to detect exon 19 deletions was able to detect > 25 types of exon 19 deletion mutations, including c.2235_2249del15, c.2237_2251del15, c.2237_2255 > T, c.2240_2254del15, c.2239_2248 > C, c.2240_2257del18, and c.2252_2276 > A (Fig. 2c); the same stuntmer was also able to detect insertion mutations (c.2234_2235insAATTCCCGTCGCTATCAA) in exon 19 (Fig. 2d).

### The detection sensitivity of stuntmers can be enhanced with nested PCRs without loss of specificity

**Fig. 3:**
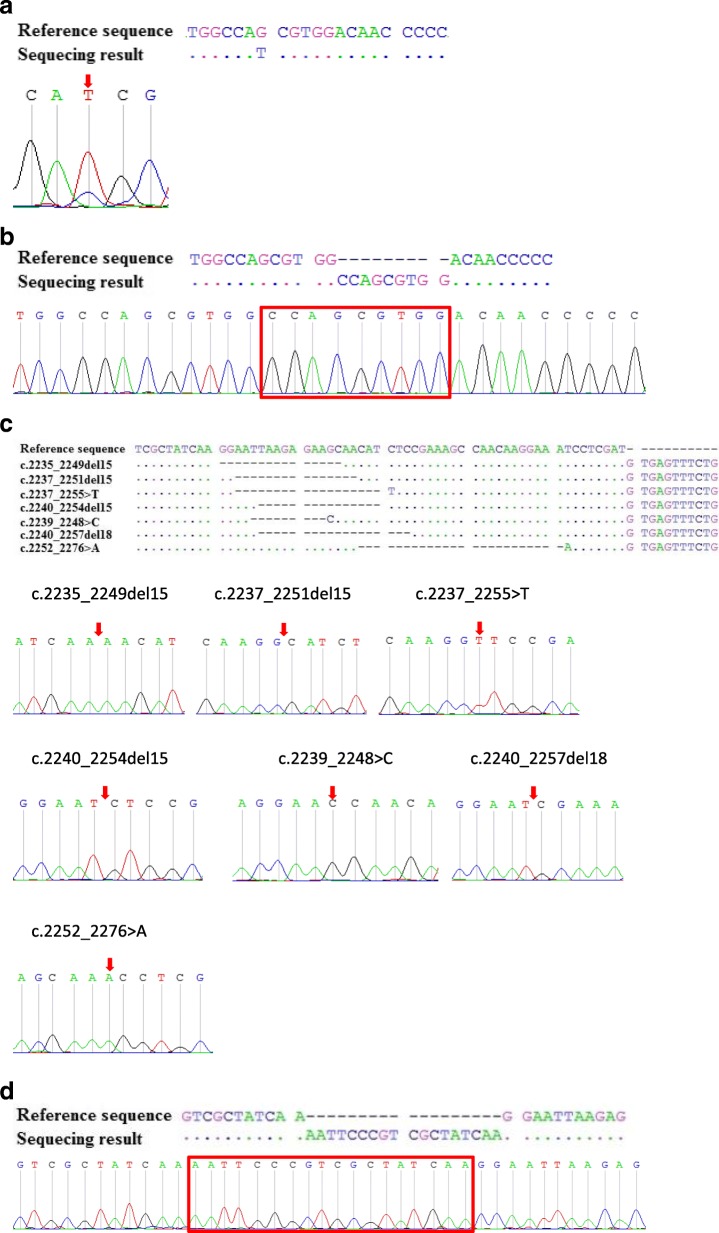
Different types of mutations in the same region can be amplified by the same stuntmer. The EGFR S768 stuntmer was able to selectively amplify the point mutation (**a**) and the insertion mutation (**b**) in EGFR exon 20. The stuntmer designed to detect deletions in exon 19 was able to detect multiple types of deletion mutations (**c**) as well as an insertion mutation (**d**). In all cases, the sequencing results indicated that the number of mutant templates exceeded the number of wild-type templates in stuntmer PCRs

Since the stuntmer has the ability to “suppress” the wild type template replicated, we hypothesis that we can increase the content of the mutant template by repeating the PCR reaction. We used the circulating free (cf) DNA Reference Standard Set (Horizon Discovery) to test if the mutation detection sensitivity of the T790 M stuntmer could be enhanced via nested PCRs without loss of specificity (Fig. 3). When the first PCR reaction is completed, we used the PCR product as a template and performed a complete PCR reaction with the same stuntmer primers to further enhance the enrichment of the mutant templates. After the primary PCR round, no mutant signal (T) was detected in the 0.1% group (where the mutant template comprised only 0.1% of the total population), and a small C peak (which corresponds to the wild-type template) was still visible in the 1% group. After the secondary PCR round, the mutant signal (T) was equal to the wild-type signal (C) in the 0.1% group, whereas the wild-type signal was completely suppressed in the 1% group. After the tertiary PCR round, only the mutant signal appeared in the 1% group, and the mutant signal was stronger than the wild-type signal in the 0.1% group. Furthermore, even after three rounds of PCRs, the original wild-type signal group remained unaltered, demonstrating that the stuntmer does not alter the original sequence of the sample, and that 100% positive predictive value can be maintained while increasing detection sensitivity with nested PCRs.

## Discussion

One of the most difficult challenges in developing diagnostic techniques is to increase the sensitivity of detection, while simultaneously maintaining or reducing the risk of obtaining false positives. Almost all signal amplification and type-specific detection assays have the potential risk of false positives or negatives, and in many cases, no confirmatory tests are available to support or negate such results. Since companion diagnostics for targeted therapy need to use detection methods with high sensitivity, such false positives/negatives could be disastrous as they can lead to the application of unsuitable treatment options.

In addition to the above problems, biopsies of primary tumors often do not contain sufficient genetic information to help gauge the metastatic potential of tumor cells; in such cases, cfDNA analysis of liquid biopsy specimens can provide genetic information on the presence of primary and metastatic tumor cells [36–40]. Current methods of tumor cell detection suffer from issues regarding sensitivity and specificity [36, 41–43]. Since most tests with high sensitivity may also have high rates of false positives, it is imperative to develop methods that are not only sensitive, but also accurate [41, 42, 44–46]. Stuntmer PCR provides a simple, low-cost, highly sensitive, accurate, and highly-specific platform for developing companion diagnostic tests.

The design of stuntmer and conventional primers differs in that a single primer can recognize two different sequences. The specificity of identification is enhanced by the interference of the primer itself. The main advantage of the stuntmer methodology that we describe in this study is that this technique can enhance amplification of both high- and low-frequency mutant alleles, with a focus on depressing the amplification efficiency of the wild-type allele, rather than amplifying high-frequency mutant alleles. Furthermore, since both the R and E regions of the stuntmer bind to reference sequences, the identity of the mutant allele is irrelevant in stuntmer design. This feature not only makes it easier for users to design stuntmers, but also allows a single stuntmer to be used in detecting several different types of mutations that may occur in a particular region; these include point mutations, deletions, and insertions. Another advantage of using stuntmer PCR is that since a single stuntmer primer can detect different types of mutations, the amount of biological tissue/samples required for testing is greatly reduced. In addition, stuntmer PCR procedures are similar to traditional PCRs, with a single optional difference—the use of an additional higher temperature annealing step to enhance mutant template amplification.

Although stuntmer PCRs are much more sensitive than traditional PCR in detecting mutations, there are three situations in which this technology may fail to detect mutations in a specified region: (a) if the mutations do not occur in the R region; (b) if two point mutations occur very far apart from each other and require two stuntmers for detection, there is a possibility that the two stuntmers may interfere with each other, and cannot be used together in PCRs; and (c) if the binding of the R region to the template is very strong, there is a possibility that without mutations in this region, the stuntmer will bind only via the R region, and no PCR products will be formed. The R region may also bind to the mutant template and inhibit replication. This situation may arise in wide mutation hotspots region required long R regions. However, it may be possible to bypass this particular problem by using multiple stuntmers with smaller R regions and weaker binding strengths.

In addition to all these points, stuntmer primers can also be used in other platforms, such as real-time PCR and NGS. In NGS, deep sequencing is used to disentangle subpopulations in complex biological samples [47, 48]. These include detection of mutations in FFPE or fine-needle aspiration biopsy specimens [18, 49, 50]. The most important limitation in using NGS for mutation detection analyses lies in the inability of this technique to resolve low-abundance mutations [51, 52]. However, combining stuntmer technology with NGS may help in resolving this issue.

## Conclusions

Stuntmer PCR can increase detection sensitivity without affecting specificity. A stuntmer primer set can amplify various mutations in defined regions and use only a typical thermal cycler. In the future, stuntmer primer may also be used on a variety of platforms, such as NGS and in different areas, such as detection of rare antibiotic-resistant mutations in bacterial populations.

## Methods

### Stuntmer design

**Fig. 4:**
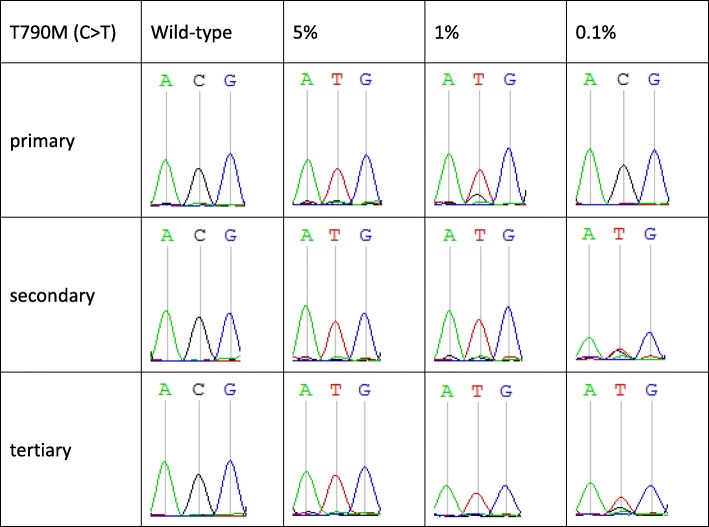
Different stuntmer detection sensitivity levels. The mutant detection sensitivity of the T790 stuntmer with the cfDNA Reference Standard Set was tested. Nested PCRs using the T790 stuntmer were able to effectively increase the mutant detection sensitivity of the stuntmer without affecting specificity. The primer successfully reversed the ratios of the mutant and wild-type sequences in the PCR products, allowing the detection of mutant templates that made up only 0.1% of the sample

A stuntmer is a primer containing three regions arranged along the 5′-end to the 3′-end in the following order: the recognition (R) region, the linker region, and the extension (E) region (Fig. 4a). The R region is designed to recognize a mutation hotspot and hybridize to the wild-type sequence. The Tm value of R region is about 60 °C to 65 °C. The E region binds to sequences upstream of the mutation site. The Tm value of E region is about 55 °C to 60 °C. About 4 to 10 bps of the 3′-end sequence of the E region overlaps with 5′-end of the R region. When the R region binds to the template, it inhibits template amplification by blocking the binding of the E region; however, if the R region does not hybridize with template, the E region is able to bind, and the stuntmer will be extended during the PCR cycle. Furthermore, if the R region does not match the template, the E region can enhance the instability of the binding. The linker functions as a connector for the E and R regions, and to terminate extension of the complementary strand.

The stuntmer can bind to the wild-type template in two different ways (Fig. 4b, c). However, because of the mismatch between the R region and the mutant template (Fig. 4d) which leads to unstable binding, the stuntmer can bind to mutant templates in only one conformation (Fig. 4e). When the R region is bound to the template, the complementary strand will not be synthesized. Ideally, every round of PCR amplification leads to the production of 2 copies of a template; if, however, the binding efficiencies of the E and R regions to the wild-type template are equal, the PCR amplification efficiency of the wild-type template will be reduced by a factor of 1.5, whereas the amplification efficiency of the mutant gene will remain unaffected.

### DNA extraction from FFPE tissue and pleural effusion samples

One thousand two hundred eighty-two FFPE and 318 pleural effusion samples from non-small-cell lung adenocarcinoma patients were collected. DNA from these samples were used in traditional and stuntmer PCRs to detect mutations in the EGFR exons 19 (deletion mutations), 20 (T790 M), and 21 (L858R). DNA from 10 μm tissue sections were obtained from FFPE tissues. Briefly, tissue sections were first dewaxed (by washing twice with xylene), then rewashed twice with 100% ethanol and dried; following which DNA was isolated using the QIAamp® DNA FFPE tissue kit (QIAGEN). DNA from pleural effusion samples were extracted using the QIAamp® DNA mini kit.

The positive percent agreement = 100% if the number of samples both traditional and stuntmer PCR found positive is equal to the number of traditional PCR-positive results; the negative percent agreement = 100% if the number of samples that both traditional and stuntmer PCR found negative is equal to the number of traditional PCR-negative results; the overall percent agreement = 100% if the number of samples with detected mutations is identical in both methods/all samples.

### Plasmid construction for sensitivity tests

Nine plasmids containing different EGFR exons, namely exon 19 wild-type, exon 19 deletion (c.2235_2249del) mutant, exon 20 wild-type, S768I (c.2303G > T), exon 20 insertion (c.2308_2309insCCAGCGTGG), T790 M (c.2369C > T) mutant, exon 21 wild-type, L858R (c.2573 T > G), and L861Q (c.2582 T > A), were constructed for sensitivity tests. These templates were amplified using traditional PCR primers, and the plasmids were constructed using the T&A cloning vector kit (RBC Bioscience). All plasmids were serially diluted from a stock containing 10^7^ copies/μl for creating sample mixtures of different percentages. All sensitivity tests were performed in triplicate and analyzed by sequencing.

### Traditional and stuntmer PCR conditions used for detection of mutations in exons 19, 20, and 21 of the EGFR gene

All PCRs were carried out in reaction volumes of 20 μl containing 0.1 μg of sample DNA, 0.2 μM of each primer (Table 2), and 10 μl of 2× Master Mix (JMR). After preheating at 95 °C for 10 min, 45 amplification cycles were carried out on an ABI 9700 Thermocycler (Applied Biosystems) under the following conditions: denaturation at 94 °C for 30 s, annealing at 60 °C for 30 s and extension at 72 °C for 30 s for traditional PCRs; denaturation at 94 °C for 30 s, first annealing at 65 °C for 40 s, secondary annealing at 57 °C for 40 s, and extension at 72 °C for 30 s for stuntmer PCRs. Amplification was completed with a final extension step at 72 °C for 10 mins. All experiments were carried out in duplicates, and patient samples were processed along with positive controls, negative controls, and reagent controls. The PCR products were electrophoresed in a 1% agarose gel (Amresco) to detect successful amplification.

**Table 2:** Primer sequences used in direct PCRs and stuntmer PCRs

Gene	Primer name	Sequence (5′➔ 3′)	Amplicon size
Traditional PCR			
EGFR exon 19	Exon 19_Forward	GCAATATCAGCCTTAGGTGCG	323 bps
	Exon 19_Reverse	AGCAGCTGCCAGACATGAGA	
EGFR exon 20	Exon 20_Forward	GAAACTCAAGATCGCATTCATG	365 bps
	Exon 20_Reverse	CAAACTCTTGCTATCCCAGGAG	
EGFR exon 21	Exon 21_Forward	CAGCCATAAGTCCTCGACGTG	399 bps
	Exon 21_Reverse	GAGCTCACCCAGAATGTCTGG	
Stuntmer PCR (see NOTE)			
EGFR exon 19 deletion			
Forward	***CCCGTCGCT***ATCAAGGAATTAAGAGAAGCAAC-C3-TAAAATT***CCCGTCGCT***		148 bps
Reverse	AGCAGCTGCCAGACATGAGA		
EGFR S768			
Forward	***TACGTGAT***GGCCAGCGTGGACAACC-C3-CCAGGAAGCC***TACGTGAT***		277 bps
Reverse	CAAACTCTTGCTATCCCAGGAG		
EGFR T790			
Forward	***ACCGTGCA***GCTCATCACGCAG-C3-CTCACCTCC***ACCGTGCA***		213 bps
Reverse	CAAACTCTTGCTATCCCAGGAG		
EGFR L858			
Forward	***GATTTT***GGGCTGGCCAAACTGCTGG-C3-AGCATGTCAAGATCACA***GATTTT***		203 bps
Reverse	GAGCTCACCCAGAATGTCTGG		

The R region: red; E region: blue; linker (C3):grey; overlapping area: highlighted in yellow

We used two annealing temperatures instead of one for the stuntmer PCR to enhance mutant-selective amplification; the purpose of using the higher annealing temperature was to enhance the binding specificity of the R region to the wild-type templates. However, stuntmer PCRs carried out using a single annealing temperature were also able to amplify mutant templates even if the mutant prevalence was only 1% in the samples (Additional file 1: Figure S1).

### Sanger sequencing

All PCR products were cleaned using the illustra ExoProStar 1-Step™ kit (GE Healthcare Life Sciences). Sequencing of forward and reverse strands (for PCR products from traditional PCRs), and only reverse strands (for PCR products of stuntmer PCRs) was done using the ABI Cycle-sequencing kit v. 3.1 (Applied Biosystems). DNA sequencing was performed using an ABI 3730 Genetic Analyzer (Applied Biosystems).

## Additional file

Additional file 1: **Figure S1.** The mutant detection sensitivity of single annealing temperature. The mutant detection sensitivity of single annealing temperature with the cfDNA Reference Standard Set was tested. In exon 19 deletion, the stuntmer was able to detect the mutant templates in only 0.1% of the tested samples. The detection sensitivity of the L858R and T790 M is 1%. (DOCX 81 kb)

## Data Availability

All data generated or analysed during this study are included in this published article and its supplementary information files.

## Abbreviations

cfDNA: circulating free DNA
EGFR: Epidermal growth factor receptor
FFPE: Formalin-fixed, paraffin-embedded
NGS: Next generation sequencing
