# Correction to: A new primer construction technique that effectively increases amplification of rare mutant templates in samples

**DOI:** 10.1186/s12896-019-0562-2

**Published:** 2019-11-12

**Authors:** Jr-Kai Huang, Ling Fan, Tao-Yeuan Wang, Pao-Shu Wu

**Affiliations:** 1 Department of Pathology, Mackay Memorial Hospital, Taipei, Taiwan; 2 Department of Nuclear Medicine, Chang Gung Memorial Hospital, Taoyuan, Taiwan; 3 Mackay Junior College of Medicine, Nursing, and Management, Taipei, Taiwan

**Correction to: BMC Biotechnol (2019) 19:62**

**https://doi.org/10.1186/s12896-019-0555-1**

Following publication of the original article [1], the author informed us that the legend for Fig. 2 was incorrect.

Fig. 2 legend has been corrected in the original article.

The correct legend for Fig. 2 is given below:

Mutant sequence enrichment using stuntmer PCR. The abilities of stuntmers to detect four different mutation scenarios (single nucleotide mutation, two different single nucleotide mutations, and a deletion mutation) were tested under three different sample conditions (only wild-type, 90:10 ratio of wild-type:mutant, and 99:1 ratio of wild-type:mutant). In all cases, the stuntmers were able to detect the mutations even when the mutant templates were present in only 1% of the tested samples. The wild-type template was completely inhibited in the T790 M point mutation (**a**) and exon 19 deletion mutation (**b**). The arrow indicates where the deletion mutation occurred
