# Supplementary figures and images for: A new primer construction technique that effectively increases amplification of rare mutant templates in samples
Source: BMC Biotechnol. 2019 Aug 23;19:62. doi: 10.1186/s12896-019-0555-1 (PMC6708177; doi:10.1186/s12896-019-0555-1)

|  | Wild type | 5% | 1% | 0.1% |
| --- | --- | --- | --- | --- |
| Exon 19 deletion | 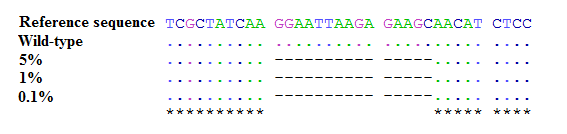 | | | |
| 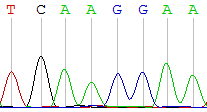 | 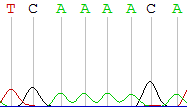 | 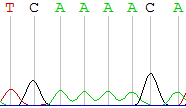 | 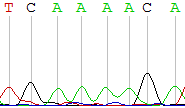 |
| L858R | 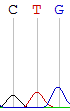 | 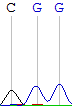 | 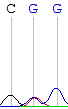 | 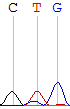 |
| T790M | 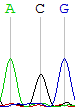 | 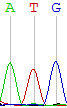 | 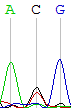 | 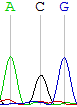 |

Supplement: Supplementary file 1 — Figure S1. The mutant detection sensitivity of single annealing temperature. The mutant detection sensitivity of single annealing temperature with the cfDNA Reference Standard Set was tested. In exon 19 deletion, the stuntmer was able to detect the mutant templates in only 0.1% of the tested samples. The detection sensitivity of the L858R and T790 M is 1%. (DOCX 81 kb) [file 12896_2019_555_MOESM1_ESM.docx]
